# Role of funders in embedding tests in conservation practice

**DOI:** 10.1111/cobi.70309

**Published:** 2026-05-06

**Authors:** Rebecca K. Smith, Nancy Ockendon, Nida Al‐Fulaij, Alessandro Badalotti, Rachel Beattie, James Byrne, Sophia Cooke, Tiago de Zoeten, Winifred F. Frick, Stephan M. Funk, Douglas Gibbs, Lauren Gupta, Tatiana Hendrix, Becky Holmes, Nicola Johnstone, James Kinghorn, Simon Mickleburgh, Florence Miller, Matthew Muir, Jacob R. Owens, Danni Parks, Victoria Reilly‐Pinion, Hannah Reid, Kevin Seely, Julien Semelin, Taylor Shaw, Ruth D. Swetnam, Lisa Wheeler, William J. Sutherland

**Affiliations:** ^1^ Conservation Science Group, Department of Zoology University of Cambridge Cambridge UK; ^2^ Cambridge Conservation Initiative Endangered Landscapes & Seascapes Programme Cambridge UK; ^3^ People's Trust for Endangered Species London UK; ^4^ Fondation Segré Geneva Switzerland; ^5^ Biodiversity Challenge Funds NIRAS Penicuik UK; ^6^ Ecological Restoration Fund London UK; ^7^ Environmental Funders Network Aylesbury UK; ^8^ Mossy Earth Earnley UK; ^9^ Bat Conservation International Austin Texas USA; ^10^ Department of Ecology and Evolutionary Biology University of California Santa Cruz California USA; ^11^ Lemu Santiago Chile; ^12^ Biodiversity Challenge Funds Department for Environment, Food and Rural Affairs London UK; ^13^ The Helvellyn Foundation Crewe UK; ^14^ International Affairs U.S. Fish and Wildlife Service Falls Church Virginia USA; ^15^ Minderoo Foundation Perth Western Australia Australia; ^16^ The Rufford Foundation London UK; ^17^ Los Angeles Zoo & Botanical Gardens Los Angeles California USA; ^18^ The Whitley Fund for Nature London UK; ^19^ Cartier for Nature Cartier Philanthropy Meyrin Switzerland; ^20^ Climate and Evidence Team The Wildlife Trusts Newark UK

**Keywords:** conservation, donors, evaluation, evidence, experiments, funding, grants, learning, monitoring, philanthropy, testing, trials, Aprendizaje, conservación, donantes, ensayos, evaluación, evidencia, experimentos, filantropía, financiamiento, pruebas, seguimiento, subvenciones, 保护, 捐助者, 评估, 证据, 实验, 资助, 拨款, 学习, 监测, 慈善, 测试, 试验

## Abstract

Effective conservation practice requires decisions based on reliable and relevant evidence, but significant gaps in the evidence base exist. Incorporating well‐designed tests of the effectiveness of interventions for biodiversity in conservation projects is one of the best ways to scale up the rate of evidence generation. Funders of conservation projects are uniquely positioned to support and incentivize such tests as part of the grant‐making process. Using a structured process, as 25 conservation funders and members of Conservation Evidence, we identified means of improving the effectiveness of the work funded through the support of enhanced evidence generation. We identified 11 approaches through which funders can support embedding testing, ranging from encouraging inclusion of a test in the project proposal, to designating separate funds for incorporating a test, to supporting an external testing unit to provide training and advice. The appropriate approaches vary depending on the scale, process, and type of funding. Barriers to supporting testing and potential solutions were discussed, barriers such as a lack of skills to check tests are appropriate and well designed and the additional time needed to develop, implement, and see outcomes of tests and differing types of projects. By encouraging testing of actions as part of conservation practice (where appropriate), funders will ultimately facilitate a stronger evidence base for more effective decision‐making and better outcomes for nature and society.

## Abstract (es)

El papel de los financiadores en la integración de las pruebas en la práctica de la conservación

**Resumen:**

Para que las prácticas de conservación sean eficaces, es necesario tomar decisiones basadas en datos fiables y pertinentes; sin embargo, existen importantes lagunas en la base empírica. La incorporación de pruebas bien diseñadas sobre la eficacia de las intervenciones en materia de biodiversidad en los proyectos de conservación es una de las mejores formas de acelerar el ritmo de generación de datos. Los financiadores de proyectos de conservación se encuentran en una posición privilegiada para apoyar e incentivar dichas pruebas como parte del proceso de concesión de subvenciones. Mediante un proceso estructurado, con 25 financiadores de la conservación y miembros de Conservation Evidence, identificamos formas de mejorar la eficacia del trabajo financiado mediante el apoyo a una mayor generación de evidencia. Identificamos 11 enfoques a través de los cuales los financiadores pueden apoyar la integración de pruebas, que van desde fomentar la inclusión de una prueba en la propuesta del proyecto, hasta destinar fondos específicos para incorporar una prueba, pasando por apoyar a una unidad de pruebas externa para que proporcione formación y asesoramiento. Los enfoques adecuados varían en función de la escala, el proceso y el tipo de financiación. Entre los obstáculos para apoyar las pruebas y las posibles soluciones se incluyen la falta de competencias para comprobar que las pruebas sean adecuadas y estén bien diseñadas, así como el tiempo adicional necesario para desarrollar, implementar y observar los resultados de las pruebas y los diferentes tipos de proyectos. Al fomentar la realización de pruebas de las acciones como parte de la práctica de la conservación (cuando sea apropiado), los financiadores facilitarán, en última instancia, una base empírica más sólida para una toma de decisiones más eficaz y mejores resultados para la naturaleza y la sociedad.

## 资助方在保护实践中纳入实验的作用

**【摘要】** 有效的保护实践需要基于可靠且相关的证据做出决策, 但现有证据基础仍存在明显空缺。在保护项目中纳入针对生物多样性干预措施有效性的精心设计的实验, 是加快证据生成速度的最佳途径之一。保护项目资助方处于独特的位置, 能够在拨款过程中支持并激励此类实验。我们作为25家保护资助方及“保护证据(Conservation Evidence)”组织的成员, 通过结构化流程, 确定了如何加强证据生成以提升受资助项目成效的途径。我们确定了资助方可以支持在保护实践中纳入实验的11种方法, 包括鼓励在项目申请中纳入实验、划拨专项资金用于整合实验, 以及支持外部实验机构提供培训和建议等。具体采用何种方法取决于资助的规模、过程及类型。支持实验面临的障碍及潜在解决方案包括:缺乏评估实验是否恰当且设计良好的专业技能, 缺乏开发和实施实验并观察其结果所需的额外时间, 此外不同类型的项目之间也存在差异。资助方通过鼓励在适当情况下将行动实验作为保护实践的一部分, 最终将有助于建立更坚实的证据基础, 从而实现更有效的决策, 并为自然与社会带来更好的保护成效。**【翻译:胡怡思;审校:聂永刚】**

## INTRODUCTION

There is an urgent need for effective conservation action in the face of the current biodiversity crisis. Evidence‐based decision‐making is vital to ensure that valuable, often limited, resources are not wasted on potentially ineffective action (Salafsky et al., 2019; Sutherland, 2022a, 2022b; Sutherland et al., 2004). Over recent decades, there have been serious efforts to collate scientific evidence of the effectiveness of conservation actions to ensure that it is easily accessible to decision makers (Pullin & Stewart, 2006; Sutherland et al., 2004). Such efforts have revealed significant gaps in the evidence base for many conservation actions, taxa, and geographical locations (Christie et al., 2019, 2020; Junker et al., 2020; Taylor et al., 2019; Tuttle & Donahue, 2022) and that tests of the effectiveness of actions are frequently based on relatively weak study designs (e.g., after designs), which can produce misleading results (Allen et al., 2023; Christie et al., 2019, 2021). By *testing*, we mean trials that evaluate the effectiveness of one specific action within a project (rather than the project as a whole, which usually involves many actions). Thus, much of conservation involves implementing actions where the evidence base is poor or weak. Increasing the rate of evidence generation, the rigor of study designs applied in conservation, and the publication of results is vital to ensure that more reliable and relevant evidence is available to decision makers and thus to enable more effective decisions resulting in better outcomes for biodiversity.

Regularly embedding testing in conservation practice (and sharing results) can act as an accelerator for generating evidence at the scale needed (Ockendon et al., 2021). Support for this change needs to come from all conservation sectors (nongovernmental organizations, local and national government, funders, business, academics, and others), but funders are particularly important in determining the allocation of funds across and within projects and so have unique influence. Grants for conservation projects come from a range of sources, including public funding, philanthropy, and private corporate funds. All these types of funders have a potential role in supporting the integration of tests in conservation practice, although philanthropic trusts and private finance may have more flexibility and agility to change their processes rapidly to incentivize this.

Funders can have a key role in increasing the rate of evidence generation by creating expectations for integration of well‐designed tests of actions, where appropriate (see below “Lack of familiarity with the process of identifying opportunities for informative tests”), and dissemination of results of projects they fund and by providing the resources and support needed. Some funders and practitioners are already committed to supporting and carrying out tests of actions (Tinsley‐Marshall et al., 2022). These funders support allocating 1–3% of grant funding to testing (Tinsley‐Marshall et al., 2022). Although this may appear a small percentage, with hundreds of millions of dollars spent annually on conservation (United Nations Environment Programme, 2022), this could still provide a substantial budget dedicated to testing, particularly for topics where existing evidence is weak (e.g., anti‐wildlife trafficking [Miller et al., 2025]). Funders are also key beneficiaries of an improved understanding of what does and does not work (including innovative approaches), allowing more efficient use of the funds they disburse and ultimately enabling improved conservation outcomes for the benefit of nature.

Support for testing complements a recent move among funders to ask applicants to reflect on evidence of the effectiveness of actions as part of grant application processes (Al‐Fulaij et al., 2025; Parks et al., 2022). Considering the evidence base should improve the design and outcomes of conservation projects and may also help applicants and funders identify where tests are needed to expand the existing evidence base. Supporting the generation of high‐quality evidence to ensure effective decision‐making is the natural next step for funders. Although this may be a relatively new concept for some funders, our aim was to provide guidance. We considered how funders that support conservation organizations in carrying out interventions can also facilitate improving knowledge of the effectiveness of actions to contribute to the wider evidence base. Separate, although important, issues are the monitoring of progress and the evaluation of programs as a whole (rather than specific conservation actions carried out as part of a wider program [Ockendon et al., 2023]) and conservation underpinned by Indigenous knowledge and approaches rather than testing, as was our focus here (e.g., Alexander et al., 2021; Tengö et al., 2014).

We, as 25 representatives of 18 environmental funding organizations, the Environmental Funders Network, and Conservation Evidence, met to develop a set of possible approaches to embedding tests of effectiveness in funded projects. We also met to identify barriers to including tests in funding requirements and potential solutions to those barriers, including lessons learned by those who have already trialed or developed processes for supporting effectiveness testing in funded projects. Our objective was to identify and share means by which we and other funders can catalyze the adoption of testing with the goal of delivering more evidence‐based practice and therefore better outcomes for nature.

## DIFFERENT APPROACHES FOR INTEGRATING TESTS IN FUNDING ALLOCATIONS

**FIGURE 1:**
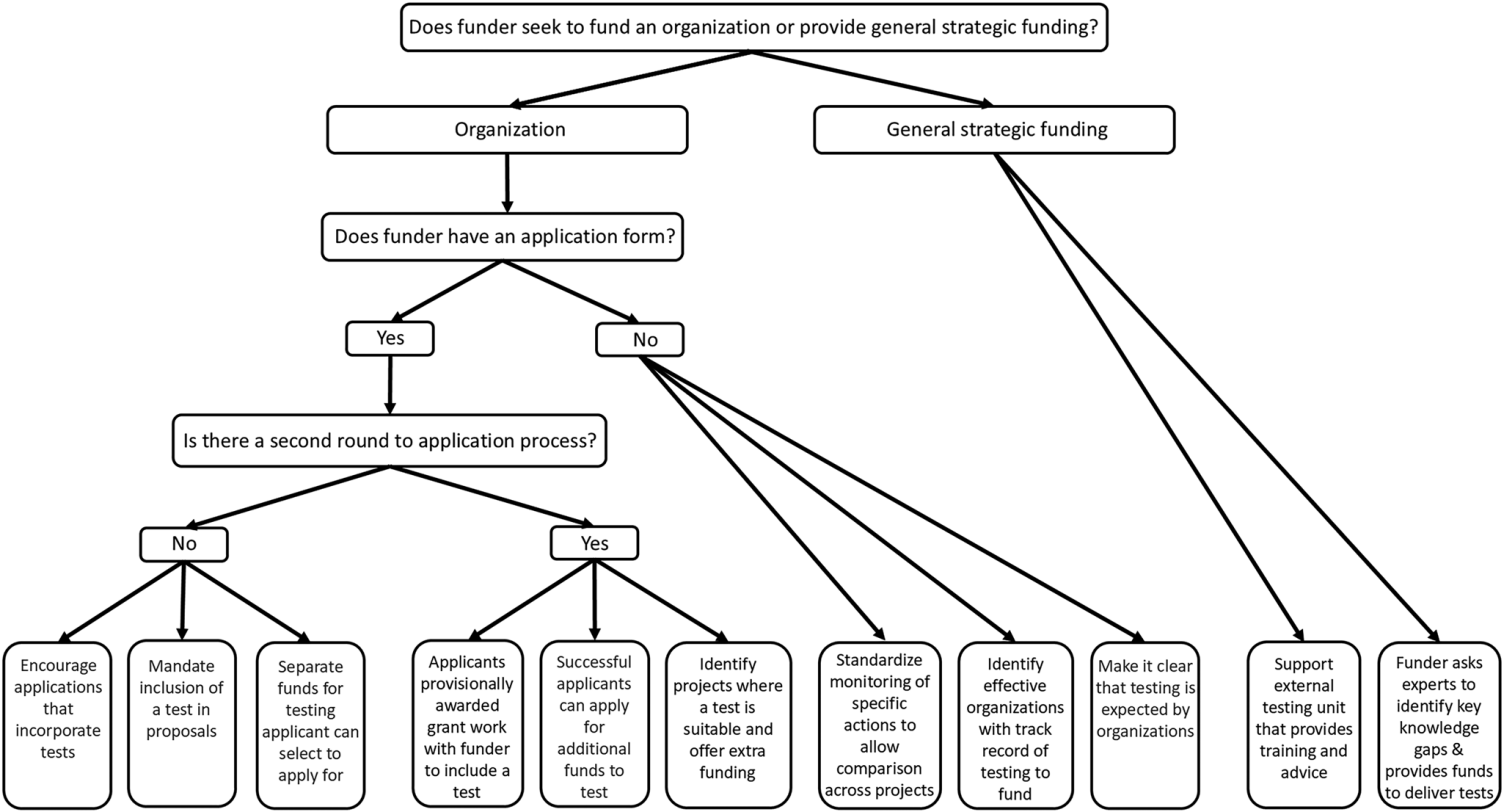
Decision tree for funders to identify which approach they could use to encourage integration of tests of actions in projects they fund (see Table 1 for strengths and weaknesses and when each is appropriate).

Initially, we created a list of approaches that are or could be used by funders to integrate tests of actions in funded projects. That, and reflections presented by five funders who already encourage testing within funded projects, formed the basis of further group discussions that resulted in a final list of 11 approaches (Figure 1). These ranged from designating separate funds that applicants can apply to for incorporating a test (e.g., Mossy Earth Innovation Grant Programme) to asking applicants who are provisionally awarded a substantial grant to further develop their project proposal with the funder to include a test before final acceptance (e.g., Endangered Landscapes & Seascapes Programme). In three separate groups, we then identified the main strengths and weaknesses of each approach and suggested under what circumstances they might be most appropriate; these are summarized in Table 1. These approaches depend on the scale, process, and type of funding. Regardless of the approach, the responsibility is on funders to ensure that incorporating tests in project proposals does not create significant amounts of extra work for applicants before funding is secured.

**TABLE 1:** Strengths and weaknesses and possible uses of the 11 different approaches that could be used to integrate tests of conservation actions in funded projects.

Approaches of funder	Potential use and strengths	Possible weaknesses
Encourage applications that incorporate tests and state this increases the chance of success; include costs in the overall budget, e.g., People's Trust for Endangered Species	Easiest and most generally effective approach that encourages testing without mandating it	May encourage devising of tests where they are not appropriate or where application is unlikely to be successful, wasting applicant's time; may discourage applications
Mandate inclusion of a test in proposals; include costs in the overall budget	Encourages widespread consideration of testing; most suitable for large projects that are likely to contain a testable element or for projects for which funds are restricted to a specific activity, such as single species management	Could discourage otherwise useful proposals if testing is impractical; may encourage devising of tests where they are not appropriate
Allocate separate funds for testing; make request for testing funds an optional part of the application form	Allows those who wish to test to do so but does not create a barrier where testing is impractical or when the applicant is reluctant to test; may require adjustment of text and finances to deliver an appropriate level of submissions	Additional funding may encourage tests that are not well integrated with the rest of the project; application for testing funding may be low
Applicants provisionally awarded a substantial grant further develop their project proposal with funder to include a test before final acceptance, e.g., Endangered Landscapes & Seascapes Programme	Most suitable for large projects with extensive project development period supported by the funder; does not waste effort of unsuccessful applicants; funders may be able to help codesign tests	Requires capacity from both funders and project teams; cost of test may not be known when grant is awarded, which may require flexibility in budgets
Successful applicants given opportunity to apply for additional funds to carry out a test	Funders can focus effort and potential funds on those where a test is realistic; need to make adding a test to a funded project attractive either because of the additional funding or because applicants understand the benefits of testing	Additional stage could result in delays to project
Identify projects where a test seems useful and practical and then approach grantee with offer of extra funding, e.g., Mossy Earth	Focus on testing where it makes the most sense; shares the burden between funders and grantees; most suitable where more resources, expertise, or access to experts who have knowledge of evidence gaps exist in the funder organization	Time‐consuming for funder with potential financial implications, e.g., on staff time; often funder wants applicant to take the lead, rather than setting agenda
Standardize monitoring of specific actions to allow comparison across projects (i.e., testing takes place at program rather than project level)	For activities that are usually carried out one at a time (e.g., reintroductions) where combining studies could allow comparison of the effectiveness of different designs; could be a powerful approach but needs standardized monitoring and a process for ensuring all outcomes are documented, not just successes	Requires a large number of comparable projects and standardized monitoring, both of which are challenging to achieve
Identify effective conservation organizations with a track record of testing actions and provide unrestricted funds	Encourages effective practice and testing when appropriate; requires evidence of testing in application form; needs clear messaging of what is expected by funder; most suitable for funders that fund organizations	May not result in a test with each round of funding
Make clear that testing is expected by organizations where appropriate to encourage applicants to consider this when planning and applying for grants	Encourages cultural change by supporting those with an interest in designing and including testing; appropriate when providing unrestricted funds to organizations rather than for specific projects	May be difficult for the funder to assess whether grantees are meeting these requirements
Identify a topic, ask a group of experts to identify key knowledge gaps and testing opportunities, and then provide funding to deliver tests, e.g., U.S. Fish and Wildlife Service was considering this	Group of experts identifies gaps, priorities, and options; most suitable for a funder or group of funders who have focused interest in a subject area	May be challenging to organize; risks creating competition for funding rather than collaboration
Support external testing unit to provide training and advice and suggest tests alongside other options	Suitable for funders wishing to improve effectiveness in a field rather than for a specific project; external group, specializing in identifying opportunities and designing experiments, can provide advice during project planning and link to topic or method specialists where needed; such a unit could in principle facilitate sharing of standardized methods, guidance, and lessons learned, helping to create efficiencies and enable networking; unit must work collaboratively and enable capacity building with practitioners working on the ground	Unit likely to require separate funding; risk of top‐down approach

Providing a range of different approaches allows funders to choose one that encourages an appropriate level of testing (not all projects are suitable for testing), depending on the size and number of grants that they provide. For example, a funder that provides many small grants may require a process that ensures that just a small proportion of the projects funded include a well‐designed test, whereas a funder that provides fewer larger grants, each of which comprises multiple activities, may require that at least one of the actions associated with each grant is tested. Alternatively, where unrestricted funds are provided to organizations rather than grants for specific projects, the funder could state that testing, where appropriate, is encouraged or expected, resulting in at least one test.

## BARRIERS AND SOLUTIONS TO INCORPORATING TESTS OF ACTIONS IN FUNDING ALLOCATIONS

Although there are a number of different approaches that funders could use to encourage tests within projects, as outlined above, there are a range of challenges and barriers that can hamper this. As a group of 25 funders, we identified these barriers and possible solutions to overcome each. Here, we describe the eight main challenges raised and how those can be addressed.

### Lack of familiarity with the process of identifying opportunities for informative tests

It may be novel for funders to support the inclusion of tests of actions in project proposals, and they may lack experience in identifying appropriate opportunities. Tests should be encouraged only when they are appropriate and useful. Therefore, to address the barrier, a set of questions could be used, as part of the application process, to help identify the projects where this is the case (i.e., to identify the action, need for the test, and design and resource requirements of the test). Table 2 provides modified questions used in the Endangered Landscapes & Seascapes Programme proposal template for applicants along with a case study. Table 3 provides a checklist that could be used by funders; all boxes would need to be checked. Open Standards for the Practice of Conservation (Conservation Standards) (Conservation Measures Partnership, 2025), an approach for project planning that includes developing theories of change and objectives, can also help determine whether tests should be done, what data are required, and how it will be analyzed and shared.

**TABLE 2:** Possible questions for conservation grant applicants to answer to identify whether a test is appropriate.

Question	Example from Cairngorm Connect Endangered Landscapes & Seascapes Programme
What is the exact action to be tested?	Impact of clearing ground vegetation on rates of natural tree regeneration will be tested.
What difference will knowing the outcome of the experiment make to the future effectiveness of your management?	The funded organization currently undertakes planting of tree seedlings across large areas, which is expensive and time‐consuming. Natural regeneration rates are currently observed to be low. However, if clearing ground vegetation substantially increases rates of natural woodland natural regeneration, this would allow expansion of forest restoration at a lower cost.
Have you checked the existing evidence? Has the experiment been carried out previously?	Some evidence is available that suggests it might work, but it is not specific to the region and habitat type in question.
Will there be a control or counterfactual, or a comparison? Provide details.	Surrounding areas will be left uncleared. Matched, uncleared plots next to the cleared plots will be monitored for natural regeneration rates without clearing.
Will there be replication? If so, provide details.	Six 20 × 20‐m plots will be cleared across a large landscape. Six matched 20 × 20‐m control plots will also be monitored.
How will it be carried out?	Using brush cutters to clear the ground in the cleared plots in the first winter of the project. All vegetation and brush will be removed from the area.
What extra time is needed to carry out the test of the action, in addition to routine management?	Half a day of work plus machinery for brush cutting.
What data will be collected, when, and by whom?	Number of seedlings will be counted in the 12 plots. One survey (approximately 3 days) every 2 years by project ecologist.
What analysis will be done and by whom? How will this be documented and by whom?	Number, height, and species of seedlings detected in cleared and uncleared plots will be compared. Results will be published after approximately 6 years in a practitioner‐focused journal.
What extra time is needed to analyze and document?	Two weeks of staff time.
Total extra time and resources	Half‐day brush cutting, 6 days by project ecologist to monitor, 1 day each for ecologist and manager to plan, 2 weeks to analyze and document, 22.5 days in total. Also need to have a brush cutter.

**TABLE 3:** Checklist for funders to identify whether a test is appropriate within a project.

Has an action that can be tested been identified?
Is it important enough to warrant a test?
Has evidence been checked to determine whether the question has already been answered?
Is better evidence needed?
Does the uncertainty warrant a test?
Is it possible to carry out a reasonably well‐designed test?
Does the team have access to the resources and skills needed to carry out the test and analyze results?
Are there plans for how results will be shared?

Often, it will not be relevant to include a test. For example, testing is not necessary if sufficient evidence already exists for the proposed action, so a first step is to check the existing evidence base (e.g., using Conservation Evidence [Sutherland et al., 2019]). Where limited or no evidence exists, value of information methods can be used to evaluate the benefit of resolving uncertainty by testing compared with acting (Bolam et al., 2018; Cenessa et al., 2015). Testing should only be undertaken where it is feasible to employ a robust study design, which allows statistical evaluation of effectiveness. It is vital to include a comparison of some kind (e.g., untreated control, before–after comparison, comparison of two different treatments, ideally with replication in each case). Counterfactual impact evaluation methods require that treatments and untreated controls are assigned randomly or statistical adjustments are made to control for confounding factors (e.g., Baylis et al., 2016; O'Garra et al., 2025).

It is only appropriate to include a test when resources or skills are available (or can be acquired) to monitor the required outcomes over the appropriate timescale or to analyze and share results. Also, testing should generally only be considered when the effect of single components or actions can be measured. Determining the effect of an action is likely to be challenging if a combination of many actions is implemented. In addition, some actions may be harder to test, such as those aimed at systemic change (e.g., some long‐term policies and international agreements), but these may be especially important to test if the necessary expertise is available (Ferraro et al., 2023). For the reasons outlined, testing will not be part of many projects, but if well‐designed tests are undertaken when appropriate and the results are published, then collectively practitioners are generating a more comprehensive evidence base for the benefit of the community as a whole.

### Lack of capacity and expertise to support tests

Funders often lack the expertise to check study designs for tests. There are a range of possible ways to improve capacity and skills, including provision of improved guidance on designing and implementing effective tests for funders and for grantees who currently often lack the capacity to develop, implement, and report on tests. Such technical assistance could be provided (e.g., by testing units, see below) in the form of videos, written resources, one‐to‐one support, workshops, facilitated peer‐to‐peer workshops and learning, online cohorts that can support each other, or online training courses. For example, the People's Trust for Endangered Species arranged for Conservation Evidence to present online webinars about evidence use, the importance of testing actions, and how to design tests for applicants before they submitted their proposals, including time for specific questions about their areas of work. Guidance on analyzing and sharing results is also needed to ensure others learn from findings. The *Conservation Evidence Journal*, which was created specifically for practitioners to share results of tests, has a template article that can be used to help submit results. It is important that such resources are equitable and made easily accessible to enable capacity building across the globe.

Sharing resources and guidance among funders could reduce the need for each funder to develop their own processes to ask for tests in proposals. For example, Table 2 provides a set of questions that could be used or adapted by funders to check study designs for tests as part of their application process (e.g., Will there be a control or counterfactual or a comparison? Will there be replication? What data will be collected? What analysis will be done?).

Other options include funders establishing collaborations with or getting help from academic researchers or experienced practitioners to design tests and potentially analyze data (additional time may be required) or creating a mentor or alumni network. Alternatively, external support could be provided through the creation of testing units of researchers and scientists who provide training, guidance, and advice on suitable tests, robust study designs, analysis methods, and open‐access sharing of results. A relevant example from a different discipline is the Independent Statistical Standing Committee for Research into Huntington's Disease (CHDI Foundation, n.d.), which is supported by a funder. Providing standardized training and guidance could help create efficiencies and enable valuable networking. We highlight the importance of such groups working collaboratively and enabling capacity building for practitioners working on the ground.

### Time needed to develop projects that include testing

Incorporating and designing tests may require extra time and support during proposal development. Some additional coordination may be required between funder and practitioner organizations to incorporate tests. For Mossy Earth, the extra coordination needed has not been costly in terms of funder staff time, but it has sometimes delayed project timelines due to back‐and‐forth communications to refine plans. One possible solution is to use questions, such as those provided in Table 2, as part of the application form when a project is being formulated. An alternative model is one in which the design and development of the testing component of a project are an ongoing, collaborative effort between funder and grantee, which would not necessarily hold up project contracts or the release of funds.

### Time needed for implementing tests

There is concern that tests often require substantial extra time and effort and hence resources to set up, monitor, analyze, and report data. Although some tests may require substantial extra effort to set up and monitor, many others may be embedded in the running of a project relatively easily. In many cases, well‐designed tests can be carried out on small budgets. Sutherland, Robinson, et al. (2022) list 100 tests of actions that could be completed within less than a month of fieldwork. For example, testing a modification to an existing treatment for controlling invasive plants by using different treatments on similar areas and monitoring survival or growth in each or comparing occupancy rates of different designs of nest boxes should be relatively cheap and simple to integrate in existing or planned management. Guidance on how to design and implement effective tests should be provided to applicants so that additional resources required are factored into the project.

Funders need to balance the desire to incorporate tests in funded projects against the potential burden of asking applicants to design, undertake, and report on well‐designed tests. However, as a group of funders, we believe that extra money is not a substantial barrier to testing and suggest that providing extra funding or relaxing other reporting requirements could increase buy‐in from practitioners. For instance, going through the questions in Table 2 should enable projects to avoid tests that are not appropriate or will not provide data robust enough to learn from and to focus instead on those that are likely to yield informative results.

### Time needed to see the outcomes of tests

There is a substantial concern that it may not be feasible to test and demonstrate change within a 2‐ to 5‐year grant period. If an organization wishes to test actions where the ultimate outcome is unlikely to be seen within the funding period, one solution is to encourage applicants to monitor an intermediate or interim outcome as well as, or instead of, the ultimate outcome or goal. For example, if a project is testing the effect of different tree planting methods on bird diversity, effects will be seen more quickly on the growth or survival rate of trees than on the ultimate target of bird communities in the forest (Watts et al., 2020). Identifying such intermediate outcomes requires that evidence exists on the trajectory and linkages between intermediate and ultimate outcomes. However, where this is available, this approach allows the project to report initial results of their test to their funder within the grant period. Ideally, the project would also continue to monitor the ultimate target of the test in the long term to ensure that preliminary results lead to the expected final outcome. Alternatively, funders could consider providing additional funds for monitoring sometime after the main grant has finished. This could provide an incentive for funders to maintain relationships with grantees beyond the main grant period.

### Differing complexities of projects and activities

Different projects have varying outcomes, with simpler interventions, perhaps those working on the removal of a single threat to a particular species, being more amenable to testing compared with more complex projects involving multiple social and ecological actions and outcomes. Depending on the project context, it may be possible to isolate and test the effects of individual actions in a complex project (e.g., if a specific set of actions is being implemented multiple times). In some cases, understanding the combined effects of actions that are frequently implemented together may also be useful. However, in many cases, it is important to recognize that projects where many actions are implemented together may not be suitable for testing. We stress that it is important to distinguish attempts to measure project impact, to show how the overall project has resulted in change, from tests of the effectiveness of specific management actions (Ockendon et al., 2021). When projects include complex social outcomes and multiple actors, it is important to ensure collaborative participatory design, monitoring, evaluation, and learning (e.g., Leisher et al., 2022; Raymond et al., 2010; Tengö et al., 2014).

### Reduced focus on conservation action due to bias in project selection

Requiring or encouraging testing could result in a bias in the types of projects or organizations funded, with funds becoming focused on evidence gathering (and organizations that have such capacity) and less complex projects rather than those that might make the most difference on the ground. One way to avoid bias in project selection is to consider testing options only once decisions about project funding have been made. Alternatively, clear criteria for funding decisions can be used, and the inclusion of testing could be applied at a second stage, once a subset of projects has been selected based on their conservation outcomes (Figure 1). Including the potential for a project to include an informative test to improve its future effectiveness as one of the assessment criteria, at a proportionate scoring level relative to other, potentially more significant criteria, could allow projects and organizations that use their projects to improve the evidence base to be appropriately incentivized and recognized. These approaches also help reduce the time required to complete additional sections about testing for unsuccessful applicants (see “Time needed to develop projects that include testing”). In addition, as outlined above (e.g., “Lack of familiarity with the process of identifying opportunities for informative tests”), testing is not relevant for every project, which allows for a variety of types of projects to be funded.

### Defining or limiting the role of funders in designing and developing project activities

Funders taking a more active role in suggesting and planning tests may affect the important funder–grantee relationship. Often the funder or applicant wants the funder to remain neutral, with the applicants leading the direction of the project. Funders must, therefore, engage sensitively with the diversity of practitioners and projects that they support if these additional requirements for funding are included (Blackwatters et al., 2023). Transparency in who is deciding the priorities for a project and clearly defining the role of the funder and the grantee at the outset of a funding relationship is important. It is likely that grantees will be best placed to identify priority questions that can be realistically tested as part of ongoing management. Funders may have a role in providing (either direct or indirect) support for grantees, in terms of resources and advice, to maximize the success of their project, including any testing component.

## CONCLUDING REMARKS

Incorporating tests of actions in conservation practice needs to become much more frequent, and these tests need to be well designed and results widely shared to improve the evidence base. Funders can play a significant role in this change by creating an expectation of testing within relevant projects and ensuring this is backed up with the support and funding required. At the same time, funders will benefit from the resulting increased evidence base, enabling more efficient use of future funds. There is already agreement from some funders to support testing and from practitioners to carry out testing (Tinsley‐Marshall et al., 2022). For example, since 2018, the Endangered Landscapes & Seascapes Programme has asked all the ecosystem restoration projects that it funds to incorporate at least one test of an action in their grant proposals. This program has identified that grantees believe that these restoration trials are one of the most useful parts of the science and monitoring required by the program, saying that they provided the opportunity to collect quantitative evidence for the effects of different management practices, such as different grazing regimes, and to share their results with local farmers.

Limited evidence is a broad, system‐wide problem in conservation. A collective evidence problem may require some of the individual solutions we describe here, but it may also require more cooperation and coordination between funders and other groups to benefit the whole community and avoid inefficient resource allocations (Fehr et al., 2002; Gross & De Dreu, 2019). One possible solution is the collective identification and prioritization of evidence needs for specific conservation topics, including for less studied species or geographic locations. For example, what are the most important evidence needs to more effectively address human–elephant conflict or to restore seagrass? This approach can draw from the Conservation Evidence database, which identifies evidence gaps, and established methods in priority setting for conservation research and emerging issues (e.g., Harper et al., 2021; Sutherland et al., 2011; Sutherland, Bennett, et al., 2022). If funders can work together, as well as with practitioners and other groups, to collectively develop priorities within particular topics and commit to addressing evidence needs more routinely, then other solutions to increase the rate of evidence generation and the rigor of study design in conservation may emerge.

We have provided 11 different approaches for funders to support and incentivize testing of actions in conservation practice and outlined possible solutions to barriers. We hope this effort inspires other funders to support testing so that there will be a significant and much needed increase in the number of tests in conservation practice. Promoting opportunities to test and creating a culture in which testing is encouraged and valued will build awareness and skills and eventually make well‐designed testing of the effects of actions a standard step in the design of conservation projects. Our vision is a more robust and complete evidence base to enable more effective decisions and thus improved global conservation practice.
